# The Role of Natural Products from Herbal Medicine in TLR4 Signaling for Colorectal Cancer Treatment

**DOI:** 10.3390/molecules29122727

**Published:** 2024-06-07

**Authors:** Yan Luo, Guochen Zhang, Chao Hu, Lijun Huang, Dong Wang, Zhejie Chen, Yumei Wang

**Affiliations:** 1 School of Basic Medical Sciences, State Key Laboratory of Southwestern Chinese Medicine Resources, Chengdu University of Traditional Chinese Medicine, Chengdu 611137, China; 2020ks053@stu.cdutcm.edu.cn (Y.L.); zhangguochen@stu.cdutcm.edu.cn (G.Z.); huanglj27@cdutcm.edu.cn (L.H.); dwang@cdutcm.edu.cn (D.W.); 2 School of Pharmacy, Chengdu University of Traditional Chinese Medicine, Chengdu 611137, China; 2020zs399@stu.cdutcm.edu.cn; 3 Shanghai Key Laboratory for Nucleic Acid Chemistry and Nanomedicine, Institute of Molecular Medicine (IMM), Renji Hospital, School of Medicine, Shanghai Jiao Tong University, Shanghai 200127, China

**Keywords:** TLR4 pathway, biochemical process, traditional Chinese medicine, inhibitors, colorectal cancer

## Abstract

The toll-like receptor 4 (TLR4) signaling pathway constitutes an intricate network of protein interactions primarily involved in inflammation and cancer. This pathway triggers intracellular signaling cascades, modulating transcription factors that regulate gene expression related to immunity and malignancy. Previous studies showed that colon cancer patients with low TLR4 expression exhibit extended survival times and the TLR4 signaling pathway holds a significant role in CRC pathogenesis. In recent years, traditional Chinese medicines (TCMs) have garnered substantial attention as an alternative therapeutic modality for CRC, primarily due to their multifaceted composition and ability to target multiple pathways. Emerging evidence indicates that specific TCM products, such as andrographolide, rosmarinic acid, baicalin, etc., have the potential to impede CRC development through the TLR4 signaling pathway. Here, we review the role and biochemical processes of the TLR4 signaling pathway in CRC, and natural products from TCMs affecting the TLR4 pathway. This review sheds light on potential treatment strategies utilizing natural TLR4 inhibitors for CRC, which contributes to the advancement of research and accelerates their clinical integration into CRC treatment.

## 1. Introduction

Colorectal cancer (CRC) poses a significant global health threat, ranking high in both morbidity and mortality rates [1]. Projections suggest that there will be over 2.2 million diagnoses of CRC and more than 1.1 million related deaths by 2030 [2]. The clinical manifestations of CRC primarily involve intestinal dysfunction, marked by symptoms such as abdominal pain, abdominal distension, heightened frequency of bowel movements, discomfort in the bowel region, and rectal bleeding [3]. Known risk factors include environmental elements, food-borne mutagens, pollution, specific bacteria, and chronic inflammation playing roles [4]. CRC demonstrates a predilection for male individuals [5]. Due to the subtle nature of early-stage clinical symptoms, many patients are often diagnosed in advanced stages [6]. Advanced CRC can present with complications such as intestinal obstruction, accompanied by systemic symptoms like weight loss and anemia. Additionally, metastasis to lymph nodes, liver, lungs, and bones may occur, ultimately culminating in a fatal outcome [7]. Primary treatments for CRC encompass chemotherapy, radiotherapy, immunotherapy, surgery, and targeted therapy. However, issues such as surgical complications, chemotherapy resistance, and toxic side effects limit their clinical utility in CRC treatment [8,9].

Toll-like receptors (TLRs) are pivotal transmembrane proteins connecting innate and acquired immunity, responding to pathogen-associated molecular patterns [1]. Dysregulated TLRs contribute to inflammatory diseases, cancers, and autoimmune conditions [10,11]. Understanding TLRs’ involvement in CRC pathogenesis is essential for exploring potential therapeutic strategies [10,12]. TLR4 is a key member of the Toll-like receptor family [13]. Present in immune cells like dendritic cells (DCs) and macrophages, TLR4 is pivotal in detecting bacterial infections. Additionally, the TLR4 signaling cascade plays a role in the development and maintenance of complex structures in multicellular organisms. Moreover, TLR4 is implicated in immune activation across various cancers. Aberrant TLR4 expression is observed in CRC patients, where it is normally low [14]. Hence, a comprehensive understanding of the biological processes of TLR4 in CRC is crucial for exploring and developing effective pharmaceutical interventions for the treatment of CRC.

Traditional Chinese medicine (TCM), with thousands of years of history, is attracting attention for cancer therapy because of its unique characteristics and safety [15,16]. Numerous TCM products exhibit potent anticancer effects [17], regulating proliferation, apoptosis, metastasis, and angiogenesis. They also modulate autophagy, counteract drug resistance, maintain immune balance, and enhance chemotherapy efficacy in vitro and in vivo [18,19]. Currently, natural products from TCMs are emerging as crucial sources for drug discovery, offering potential in CRC treatment [20,21]. Natural products from TCM have been developed for targeting the TLR4 pathway in CRC treatment. For example, andrographolide [22], resveratrol [23], and berberine [24] can reduce inflammation of CRC by targeting the TLR4 signaling pathway, while baicalein [25], baicalin [26] , and decursin [27] can inhibit metastasis of CRC by targeting the TLR4 signaling pathway. However, systematically describing their specific molecular targets has proven challenging.

Previous reviews on the role of TLR4 in CRC primarily focus on its relationship with epigenetics, inflammation, and intestinal homeostasis [4]. In this review, we search the PubMed database using the keywords “TLR4” and “Colorectal cancer”. By reading the abstract, we identified and retained papers related to “biological processes” and “pharmacological mechanisms of TCMs product”. Then these papers were systematically categorized and retained, while irrelevant articles were excluded. We further emphasize the involvement of TLR4 signaling in CRC initiation and progression by elucidating its roles in various phenotypes such as proliferation, metastasis, and tumor immune microenvironment. Additionally, we highlight the potential therapeutic significance of TLR4 in CRC by summarizing the effects of TCM products, thus shedding light on the pivotal role of TCM products in treating CRC through the TLR4 pathway. The TLR4–myeloid differentiation protein-2 (MD2) protein complex serves as the initiating protein in TLR4 signaling activation and plays an indispensable role in the activation process [28,29]. We predict the ability of reported TCM compounds to target TLR4-MD2, revealing that most compounds exhibit strong binding affinity to the TLR4-MD2 protein complex. Representative compounds and their interactions with TLR4-MD2 are showcased. In summary, we focus on the TLR4 signaling pathway and its associated biochemical transduction processes in CRC. Furthermore, we have comprehensively summarized various inhibitors derived from natural products in TCM, that can suppress the TLR4 signaling pathway, showcasing their potential anti-CRC effects. We have delineated the reliability of TLR4-MD2 as a target for screening drugs to treat CRC, providing a valuable approach for drug discovery in CRC therapy. We’ve discussed the outcomes of past research and identified limitations, paving the way for future improvement and exploration.

## 2. TLR4 Pathway

**Figure 1:**
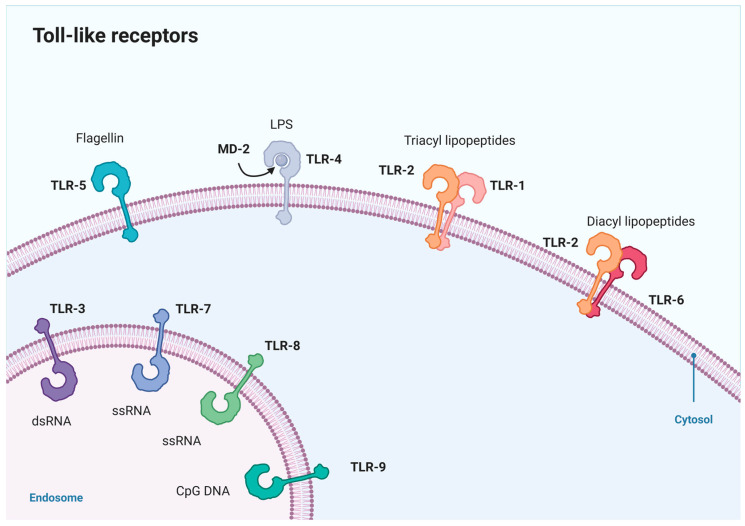
Cellular localization of mammalian TLRs. The functional activation of TLRs necessitates dimerization. In this depiction, only TLRs forming heterodimers are presented in dimeric form, while those not illustrated in dimeric state function as homodimers. (This figure was created with Biorender.com, accessed on 30 January 2024).

TLRs contribute to the immune response, detecting microbial patterns and initiating signaling pathways for pro-inflammatory gene transcription [30]. Dimerization of TLRs is a crucial mechanism for their functional regulation and a necessary step for activation, thereby initiating downstream signaling pathways and triggering immune responses. Upon stimulation, most TLRs (TLR3, TLR4, TLR5, TLR7, TLR8, TLR9) form homodimers [31], stabilized through interactions within their internal domains, which promotes the initiation of signal transduction. TLR2 primarily forms heterodimers with TLR1 and TLR6 on the cell membrane [32,33,34], expanding the signal recognition range of TLRs and regulating the specificity of their signaling pathway [31]. Ligand-induced dimerization leads to conformational changes in TLR domains, facilitating adaptor protein recruitment and downstream signaling cascade assembly. This symmetrical association ensures proper folding and stability of TLR complexes, essential for their immunostimulatory response [31]. When ligands like bacterial lipopeptides or viral RNA bind to TLR dimers, it triggers a conformational change, bringing the receptors’ TIR domains closer. This change allows the recruitment of adaptor proteins, initiating downstream signaling for the immune response. Each TLR dimer has its specificity for different pathogens, ensuring a tailored immune response while preventing overactivation [31]. Surface-bound TLRs recognize extracellular patterns, while intracellular TLRs respond to nucleic acids within endosomes [35] (Figure 1). Moreover, TLRs, notably TLR2 and TLR4, recognize not only foreign microbial ligands but also endogenous ligands termed damage-associated molecular patterns (DAMPs), Those DAMPs are released from tissue during instances of injury or inflammation [36]. Upon TLR ligand binding, receptors undergo dimerization and initiate signal cascades through adaptor proteins, including myeloid differentiation primary response gene 88 (MyD88), toll/interleukin-1-receptor-domain-containing adaptor protein (TIRAP), toll/interleukin-1-receptordomain-containing adaptor inducing interferon-β (TRIF), and TRIF-related adaptor molecule (TRAM). MyD88 is pivotal in all TLR pathways except TLR3, while TRIF exclusively interacts with TLR3 and TLR4. Activation of antigen-presenting cells, like macrophages and DCs, relies on MyD88 signaling triggered by TLR2 or TLR4 agonists. This activation triggers downstream pathways involving nuclear factor kappa-B (NF-κB) and mitogen-associated protein kinase (MAPK). Consequently, there is rapid upregulation of pro-inflammatory cytokines, chemokines, and receptors, initiating inflammation. It enhances vascular permeability, guides DCs and macrophage migration, and also regulates the development of adaptive immunity [37].

**Figure 2:**
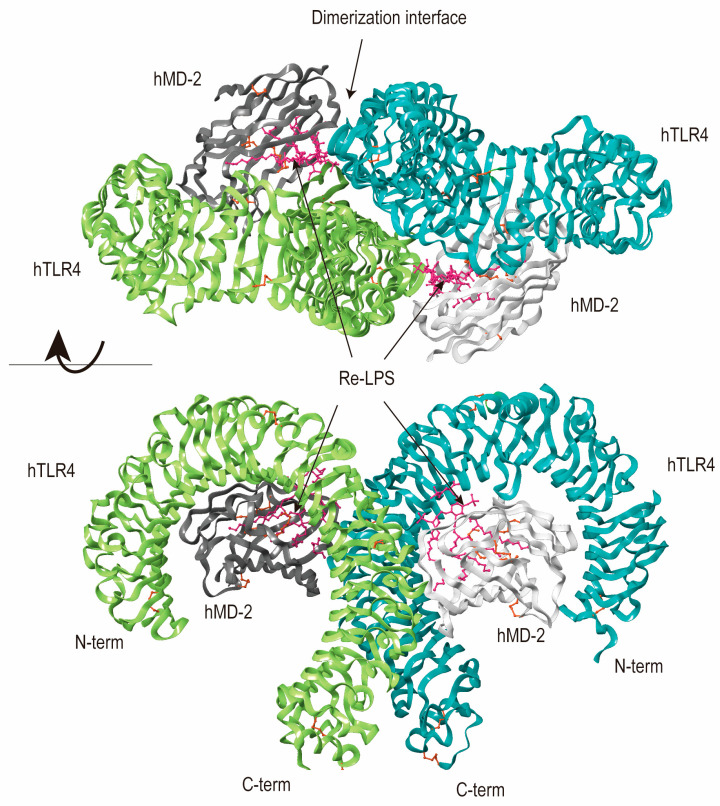
Overall structure of the hTLR4/MD2/Re-LPS complex (PDB ID: 4G8A).

TLR4, vital in sensing Gram-negative bacteria, was the first human TLR protein discovered in 1997, expressed on immune cells [38]. It stands out as a prominent pattern recognition receptor found in diverse intestinal cells, encompassing both immune and non-immune types. Its core function lies in identifying lipopolysaccharide (LPS) from gram-negative bacteria within the gut (Figure 2). Normally, TLR4 exhibits low expression on the intestinal mucosa, playing a crucial role in preserving tolerance toward commensal bacteria [39]. However, TLR4 overexpressed under specific pathogen-free conditions, documented in both human and murine models of inflammation-associated colorectal neoplasia [40].

**Figure 3:**
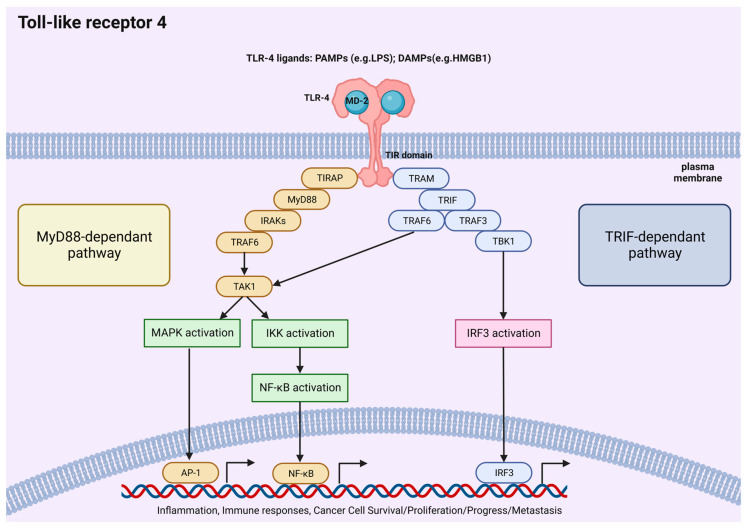
TLR4 signaling pathway. (This figure was created with Biorender.com, accessed on 30 January 2024).

The signal cascade includes different branches: TLR4 activation is initiated by LPS, with CD14 and MD2 acting as crucial proteins for LPS/TLR4 binding. Following ligand engagement, TLR4 undergoes dimerization and recruits downstream adaptor molecules, to orchestrate an inflammatory response [41]. The MyD88 pathway activates interleukin (IL)-1 receptor-associated kinases (IRAKs), transforming growth factor-βactivated protein kinase 1 (TAK1), and tumor necrosis factor receptor-associated factor 6 (TRAF6), while the TRIF pathway signals activate TRAF6/TAK1 and IKK. Moreover, the two pathways converge at NF-κB, which remains inactive in the cytoplasm when complexed with IκB. Upon proteasomal degradation, NF-κB translocates to the nucleus. Additionally, TAK1 phosphorylates MAPKs, intensifying inflammation. The TRIF pathway also triggers interferon regulatory factor (IRF)3, prompting an antiviral response. In intestinal epithelial cells (IECs), these pathways collectively maintain normal physiological functions while combating infections [4] (Figure 3). Multiple studies on microbial products and TLR4 agonists emphasize TLR4’s significant role in tumor growth, survival, and progression. Notably, LPS enhances both tumor growth and metastasis [42].

## 3. TLR4 Pathway-Related Biochemical Processes

**Figure 4:**
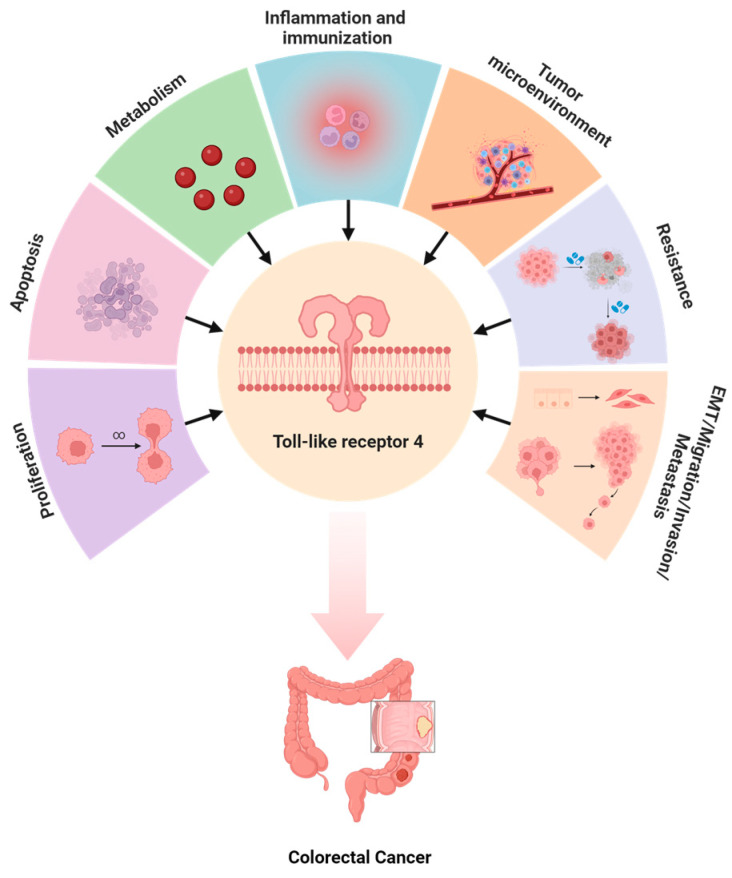
TLR4 and biochemical processes in CRC. (This figure was created with Biorender.com, accessed on 19 May 2024).

The TLR4 signaling pathway intricately interacts with various biochemical mechanisms in CRC, spanning tumor cell proliferation, apoptosis, metabolism, inflammation, immunity, microenvironment dynamics, resistance, and epithelial–mesenchymal transition (EMT), alongside migration, invasion, and metastasis (Figure 4).

### 3.1. Cancer Cell Proliferation

It is widely recognized that hyperplasia is closely linked to cancer development [22]. TLR4 is closely associated with tumor development [43]. The TLR4 pathway, a key signal cascade, is implicated in processes like proliferation, inflammation, and invasion during tumorigenesis [44]. This insight into TLR4 function is vital for developing therapeutic strategies targeting diseases, emphasizing the pathway’s significance in understanding and potentially intervening in hyperplasia-associated cancer development.

TLR4 activation by LPS induces the formation of LPS/TLR4/MD2 multimer, triggering TLR4 signaling pathways. In CRC cells expressing TLR4 and MD2, exposure to LPS leads to an upregulation of CXC chemokine receptor 7 (CXCR7) expression. The CXCR7/CXC12 axis, known for promoting angiogenesis and cell invasion, is associated with CRC cell proliferation and migration, highlighting TLR4’s role in modulating tumor cell behavior through chemokine signaling pathways [14]. In the context of cell proliferation, TLR4 regulates acyl-coenzyme A cholesterol acyltransferase 1 (ACAT1) expression in CRC cells, such as HT29 cells. Knockdown of TLR4 results in reduced expression of ACAT1, and subsequently leads to a decrease in cancer cell proliferation, highlighting the involvement of TLR4 in modulating tumorigenesis and progression [43].

**Figure 5:**
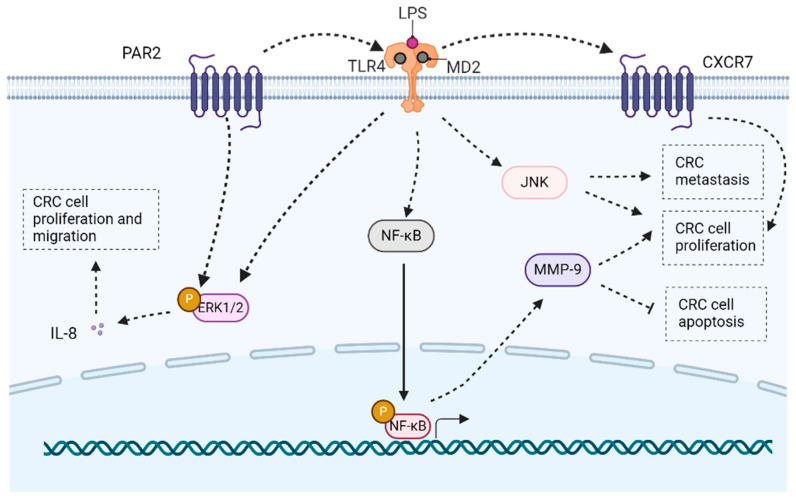
The mechanisms related to colorectal cancer cell proliferation. (This figure was created with Biorender.com, accessed on 19 May 2024).

In CRC, the aberrant activation of the TLR4/MyD88/NF-κB/MMP9 signaling pathway is pivotal in promoting cell proliferation. NF-κB, primarily in its p50/p65 form, influences key cancer processes, including apoptosis and cell cycle [45]. MMP9, a member of the MMP family, contributes to infiltration invasion by breaking down the extracellular matrix, ultimately enhancing cancer growth and dissemination in the context of TLR4 signaling [22]. TLR4 activation induces c-Jun N-terminal (JNK) activation, promoting the proliferation of CRC cells [1]. The MAPK pathway, particularly extracellular regulated protein kinases (ERK)1/2 and p38, plays a crucial role in cell proliferation, differentiation, and apoptosis triggered by TLR4 activation. Additionally, the interaction between protease-activated receptor 2 (PAR2) and TLR4 contributes to ERK1/2 phosphorylation, and influences the expression of IL-8 and transcription factors, thereby fostering cell proliferation and migration in CRC [13] (Figure 5).

### 3.2. Apoptosis

Apoptosis is pivotal in physiological and pathological contexts. It functions through intrinsic mitochondrial pathways activated by caspase-9 or extrinsic pathways initiated by specific membrane receptors responding to external stimuli, influencing inflammation, infection, and tumorigenesis [46].

Excessive reactive oxygen species (ROS) levels, induced by TLR4 activation, once surpassing a threshold, contribute to cell damage and apoptosis in tumors [47]. COX-2 experiences downregulation in intestinal tumors, correlating with elevated expression of apoptotic markers such as Bad and p21. Constitutive TLR4 signaling leads to heightened proliferation in the intestinal epithelium, accompanied by increased apoptosis, potentially mediated by the downregulation of the pivotal survival factor, COX-2 [42]. Activation of the TLR4/COX-2/prostaglandin E2 (PGE-2) pathway inhibits the secretion of anti-tumor immune cytokines, suppresses tumor cell apoptosis, and augments tumor occurrence [48].

NF-κB activation in cancer remains controversial, as it can either suppress or promote apoptosis [49]. The phosphorylation of ERK1/2 and P38 induced by LPS, alongside NF-κB activation, suggests a multifaceted role. TLR4 plays a crucial role in promoting immune escape and apoptosis resistance in human CRC cells [50].

Hyaluronic acid is produced by tumor cells and various microenvironment cells, promoting CRC growth through enhanced proliferation and motility via binding to CD44. Except for LPS, hyaluronic acid also serves as a TLR4 ligand, contributing to colorectal tumorigenesis. The interaction of hyaluronic acid with TLR4, along with CD44, activates NF-κB, leading to a decrease in spontaneous apoptosis in CRC [51]. The NF-κB and protein kinase B (AKT) pathways are involved in resistance to anti-tumor drugs and radiation by promoting apoptosis. TLR4 activation induces Akt/IKKα/β/NF-κB activation, upregulating anti-apoptotic protein Bcl-xl, contributing to apoptosis resistance in CRC cells, potentially facilitating tumor immune escape [38,52].

### 3.3. Metabolism

The symbiotic relationship between the intestinal tract and resident microorganisms is essential for preserving intestinal equilibrium. However, this equilibrium can be disrupted by changes in the microbiome resulting from environmental factors like diet, lifestyle, or infection. This disruption affects metabolism and contributes to diseases such as inflammatory bowel disease (IBD) and colitis-associated colon cancer (CAC). Beneficial bacteria engage in glycolysis and metabolize butyric acid, which impedes CRC progression. Metabolites produced by gut microbiota near the colorectal epithelium are crucial for regulating physiological processes, including immune-inflammatory responses and the transition from inflammation to cancer [24].

Short-chain fatty acids offer intestinal protection, particularly in the context of azoxymethane/dextran sodium sulfate (AOM/DSS)-induced inflammatory transformation leading to CRC. This model prominently activates the TLR4/NF-κB/IL-6/signal transducers and activators of the transcription 3 (STAT3) pathway [24]. Additionally, in CRC cells, TLR4 stimulation by LPS leads to the phosphorylation of glycogen synthase kinase-3β (GSK-3β) and relevant lipogenic enzymes. This interaction also modifies the expression of CD74, CD44, and macrophage inhibitory factor (MIF), while decreasing transcriptionally active p63 (TAp63) levels. Inhibition of GSK-3β using SB216763 leads to decreased intracellular fatty acid synthesis and modulation of CD74/CD44 expression. This underscores their potential as therapeutic targets for managing the TLR4-mediated EMT process in colon cancer cells via TAp63 regulation [53].

Aerobic glycolysis is vital for the survival and proliferation of tumor cells. In CRC, the upregulation of thrombospondin 2 (THBS2) is associated with poor prognosis. THBS2 specifically interacts with TLR4, resulting in high expression of glycolytic genes and increased glycolytic capacity in CRC cells. The THBS2/TLR4 axis elevates HIF-1a levels, contributing to glycolysis, and promoting CRC progression [54]. Moreover, palmitic acid modulates TLR4 expression. Knocking down TLR4 changes the metabolic enzyme profile, eliminating high-fat diet-enhanced ATP production and cancer growth. TLR4 acts as a master regulator in CRC growth under a high-fat diet by orchestrating cancer metabolism [55].

Elevated galectin-1 (Gal-1) expression is associated with invasiveness in CRC, and lactate production serves as a critical risk of metastasis. Stimulation with the TLR4 ligand LPS boosts Gal-1 expression, triggers EMT-related cytokines, activates glycolysis-related enzymes, and enhances lactate production in CRC cells. This TLR4/Gal-1 signaling pathway further stimulates a disintegrin and metalloproteinase (ADAM) 10 and ADAM17, enhancing invasion activity and promoting the acquisition of mesenchymal characteristics in LPS-treated CRC cells. On the flip side, blocking ADAM10 or ADAM17 effectively inhibits lactate generation and migratory capacity in LPS-treated CRC cells. Hence, the TLR4/Gal-1 pathway governs lactate-mediated EMT processes via ADAM10 and ADAM17 in CRC cells [56].

Increased ceramide levels are associated with heightened β-catenin activity and colorectal tumorigenesis, dependent on TLR4. Ceramide induces shifts in the gut microbiota, promoting colorectal tumorigenesis [57]. Arachidonic acid is synthesized by the upregulated enzyme delta-5 desaturase in CRC, regulating tumor growth through a high arachidonic acid microenvironment-induced enrichment of Gram-negative microbes. These microbes activate the TLR4/MyD88 pathway in CRC, contributing to the delta-5 desaturase-arachidonic acid axis, metabolizing to PGE-2, and ultimately encouraging tumor growth [58]. Additionally, *Fusobacterium nucleatum* infection upregulates the cytochrome P450 2J2/12,13-epoxyoctadecenoic acid axis via the TLR4/Keap1/NRF2 signaling pathway, inducing CRC development [59].

In summary, the interplay between the LPS/TLR4 signaling pathway and various metabolic pathways significantly contributes to chronic intestinal inflammation onset and intestinal cancer progression. These metabolic pathways include primary bile acid biosynthesis and secretion, peroxisome function, as well as the renin–angiotensin system, glutathione metabolism, and arachidonic acid pathways [60].

### 3.4. Inflammation and Immunization

Bacterial invasion, inflammation, and the release of inflammatory cytokines are key factors in initiating and progressing CAC. This intricate interaction entails stimulation within IECs, immune cell influx, and secretion of cytokines, resulting in inflammation, hyperplasia, dysplasia, and ultimately carcinoma formation. Enhancing comprehension of these molecular mechanisms holds the potential for novel approaches in preventing and treating CAC [61], as patients with colitis are at a heightened risk for CRC development [62,63].

*Fusobacterium nucleatum* is a risk for CRC by upregulating inflammatory mediators through potential miRNA-driven activation of TLR2/TLR4 [64]. TLR4 signaling activation induces intestinal inflammation and supports bowel cancer development [61,65]. Constitutive epithelial TLR4 activation amplifies inflammatory responses to mucosal injury, contributing to colitis-associated tumorigenesis. The regulation of TLRs influences both acute colitis and its associated cancer outcomes. Targeting TLR4 and other TLRs holds promise for preventing or treating CAC [66].

In the inflammatory phases of CAC development, increased TLR4 expression was observed in colonic tissues. Inhibiting TLR4 signaling significantly reduced colonic tumor development, accompanied by fewer infiltrating macrophages and lower pro-inflammatory cytokine levels. Bacterial stimulation of TLR4 on innate immune cells initiates inflammation, promoting tumor growth. Inhibiting TLR4 signaling during intestinal inflammation could be a novel strategy to hinder CAC development [67]. The human TLR4 gene variants Asp299Gly and Thr399Ile are linked to reduced responsiveness to inhaled LPS, affecting TLR4 signaling and potentially disrupting intestinal homeostasis, leading to chronic inflammation and, ultimately, CRC [68]. The tumor necrosis factor (TNF)-α-induced protein 8-like-2 (TIPE2), a novel inflammation-regulating protein, inhibits TLR4-mediated CRC development through caspase-8 [69].

NF-κB functions as a supervisor in regulating inflammation-induced cancer transformation, with its activation in cancer cells contributing to tumor growth. Inhibition of NF-κB, particularly through targeting the LPS/TLR4/NF-κB pathway, proves to be a promising strategy for preventing colitis-associated carcinogenesis [62]. The expression of biglycan (BGN), a TLR4 ligand, is elevated in cancers, contributing to the epigenetic silencing of siglec-7 ligands through the BGN/TLR4/NF-κB pathway in CRC cells [70]. TLRs initiate the activation of NF-κB and IRFs, determining the nature and effectiveness of innate immune responses [71]. Epithelial TLR4 activation is associated with increased dual oxidase 2 and NADPH oxidase 1 in IBD and CRC, impacting mucosal microbiota and promoting colitis-associated tumors [72]. HMGB1 activates inflammatory cytokines through TLR4, contributing to cancer, by enhancing expression via NF-κB and STAT3 signaling pathways [73].

Par3 plays a critical role in establishing epithelial cell polarity and tight junction integrity. Its deficiency allows penetration of pathogenic bacteria and endotoxins into the intestinal submucosa, activating the TLR4/MyD88/NF-κB signaling pathway. This activation promotes inflammation-driven CRC development, suggesting Par3 as a potential molecular marker for early-stage CRC diagnosis [74]. TLRs, essential recognition receptors for innate immune cells, activate inflammatory processes. The interaction between miR-155 and the TLR4 pathway involves a positive feedback loop, where TLR4 induces miR-155 expression, and high miR-155 levels augment TLR4 signaling. This feedback loop synergistically accelerates CAC development, highlighting their shared properties in promoting CRC [75,76].

Alternative splicing of mRNA contributes to immune cell differentiation and activation regulation. Eftud2, an alternative splicing factor, modulates the innate immune in macrophages. Alterations in eftud2-mediated alternative splicing affect components of the TLR4/NF-κB cascades, impairing NF-κB activation. Modulating alternative splicing in innate immune signals could be a potential therapeutic strategy for treating CAC [77]. Elevated sCD40L in the serum of patients with malignancies and IBD suggests a TLR4-sCD40L axis, potentially reducing immunosurveillance and leading to CAC development [78]. The downstream pathways of TLR4-mediated epithelial proliferation in CAC include COX-2-mediated PGE-2 production and the induction of ligands for epidermal growth factor receptor (EGFR), offering insights into mucosal prostanoid levels [79].

TLR4 plays a crucial role in inducing COX-2, elevating PGE-2 production, and activating EGFR signaling in chronic colitis, promoting CAC development. Amphiregulin, a TLR4 and COX-2-dependent EGFR ligand, contributes to EGFR phosphorylation in IECs. Inhibiting TLR4 signaling could be beneficial in treating CAC [80]. The TLR4/MyD88/NF-κB cascade is overexpressed in CAC, promoting tumorigenesis through COX-2 induction, MAPK signaling activation, EGFR activation, and pro-inflammatory cytokine production. Targeting the TLR4/MyD88 loop emerges as a potential therapeutic strategy against CAC initiation and progression [81]. Gene expression of TLR1, TLR2, TLR4, and TLR8 induces CRC-associated expression of IL-6 and IL-8 genes, serving as potential CRC markers [82]. Zfp90 accelerates CAC progression via the TLR4/ phosphatidylinositol-4,5-bisphosphate 3-kinase (PI3K)/AKT/NF-κB pathway, highlighting potential targets for treating CAC [83].

### 3.5. Tumor Microenvironment

The development and advancement of tumors depend not only on genetic alterations within neoplastic cells but also on the tumor microenvironment [84]. The tumor microenvironment is a complex system, composed of host and tumor-derived components [85]. TLR4 is predominantly expressed in immune cells like macrophages, DCs, granulocytes, and lymphocytes. Recent findings suggest the presence of TLR4 in IECs as well [86].

Tumor patients showing Cyto-HMGB1 translocation and PD-1+tumor-infiltrating lymphocytes (TILs) pre-treatment exhibit better clinical outcomes, possibly due to HMGB1 release inducing DC maturation via TLR4 activation. This attracts PD-1+TILs to the tumor site for immune scavenging. Cyto-HMGB1 and PD-1+TILs serve as predictive biomarkers for personalized oncological treatment, including immunotherapy [87]. Aberrant NF-κB pathway activation directly induces PD-L1 transcription and regulatory T cell proliferation, indicating NF-κB as a promising therapeutic target for modulating the immunosuppressive tumor microenvironment in CRC [26]. Tumor-associated macrophages (TAMs) foster tumor advancement through an immunosuppressive phenotype linked to PD-L1 expression. The TLR4/NF-κB pathway acts as a convergent mechanism for small extracellular vesicle-induced PD-L1 expression, thereby contributing to an immunosuppressive microenvironment [88].

During steady-state conditions, TLR4 is usually expressed at low levels on intestinal mucosa maintaining tolerance to commensal bacteria. However, TLR4 activation on IECs fosters a tumor-promoting microenvironment, contributing to CAC tumorigenesis [26]. Dysregulation of TLRs regarding gut microbial abundance may contribute to carcinogenesis. Furthermore, gut microbial quantity could play a role in carcinogenesis [89]. High expressions of TLR2, TLR4, and TLR5 correlate significantly with dense CD3- and CD8-positive cell infiltration, suggesting potential prognostic value for CRC patients [90].

Myeloid-derived suppressor cells (MDSCs), known for downregulating innate and adaptive immune responses, inhibit antitumor immunity by suppressing T cell functions. Activation of the TLR4/NF-κB signaling pathway triggers apoptotic cascades, selectively decreasing MDSC percentages in tumor-bearing mice spleens. This highlights the potential antitumor property of the TLR4/NF-κB pathway, elucidating a novel perspective on modulating MDSC apoptosis in vivo [46].

Macrophages, integral to the inflammatory and tumor microenvironment, are pivotal in tumor development. TAMs, play a crucial role in CRC progression, with increased infiltration correlating with disease severity. As described by Sica in 2006, TAMs exhibit M2-like characteristics, promoting immune escape and tumor growth [86]. Experimental evidence highlights macrophages’ contribution to tumor cell proliferation, invasion, metastasis, angiogenesis, and immunosuppression through cytokine secretion. During CAC, macrophages display stage-specific effects, with the M1 phenotype in colitis-premalignant stages exerting pro-inflammatory and anti-tumor effects, while the M2 phenotype in CRC stages contributes to inflammation suppression and CRC onset [86].

Heat shock protein-110 (HSP110), is overexpressed in CRC and correlates with metastasis and unfavorable prognosis. Moreover, CRC correlates with metastasis and poor prognosis in CRC patients. In CRC, HSP110 affects macrophage differentiation towards a pro-tumor, anti-inflammatory profile. Blocking HSP110 results in cytotoxic, pro-inflammatory macrophages. Extracellular HSP110’s effect on macrophages implicates TLR4. High HSP110 in CRC biopsies is associated with pro-tumoral macrophages, contributing to poor outcomes [91]. CTSK, a mediator linking gut microbiota imbalance and CRC metastasis, stimulates M2 TAM polarization via TLR4, promoting CRC invasion and metastasis through cytokine secretion. The association of CTSK overexpression with M2 TAMs and poor prognosis indicates CTSK’s potential as a significant biomarker and therapeutic target in CRC [92]. LPS triggers TOP1-binding arginine/serine-rich protein (TOPORS) via the TLR4-TRIF pathway, influencing the transcription of the tumor suppressor scaffold/matrix attachment region binding protein 1 (SMAR1) in CRC.SMAR1, an STAT3 repressor, influences tumor growth and macrophage polarization towards M1 phenotype, highlighting TOPORS as a potential regulator in CRC [93].

*Fusobacterium nucleatum* contributes to CRC development by inhibiting host anti-tumor immunity. The TLR4/NF-κB signaling pathway activation, facilitated by *Fusobacterium nucleatum*, induces S100A9 expression and M2-like macrophage activation in the CRC microenvironment. Elevated S100A9 in *Fusobacterium nucleatum*-infected CRC contributes to M2-like macrophage polarization, facilitating CRC malignancy. Targeting the TLR4/NF-κB/S100A9 cascade emerges as a promising immunotherapeutic strategy for *Fusobacterium nucleatum*-associated CRC [94]. *Fusobacterium nucleatum* also induces M2 polarization of macrophages within the CRC microenvironment through a TLR4-dependent mechanism [95]. Moreover, *Fusobacterium nucleatum* acts as a risk factor for CRC due to its potential to elevate the expression of inflammatory mediators via miRNA-mediated activation of TLR2/TLR4 [64].

DCs and natural killer (NK) cells exhibit mutual activation, mediated by TLRs on their surfaces when engaged with their ligands. In a murine model, the co-maturation of DCs and NK cells stimulated by TLR agonists shows considerable potential as an effective trigger of anti-tumor immune responses, highlighting a promising approach for developing DC-based vaccines in CRC immunotherapy [96]. Additionally, certain cancer-associated bacteria, such as Klebsiella pneumoniae, produce toxins like LPS that inhibit the p53 pathway through TLR4/NF-κB-mediated destabilization of TP53 mRNA, shaping the genomic evolution of cancer [97]. TLR4 signaling on IECs is crucial for recruiting and activating macrophages, influencing the tumor microenvironment, and promoting dysplastic lesions in CRC [98].

HMGB1 released under glucose deprivation stimulates colonic myofibroblast migration and invasion through RAGE and TLR4 activation, making HMGB1 a potential therapeutic target for inhibiting solid tumor growth [99]. Inflammatory microenvironments may contribute to CRC progression through a novel pathway, implicating TLR4-mediated NF-κB/STAT3 activation [100]. Elevated S100A9 and MDSCs in CRC patients correlate with neoplastic progression. S100A9 induces chemotaxis and activation of MDSCs through signaling pathways involving RAGE-mediated p38 MAPK and TLR4-induced NF-κB, highlighting their potential as markers for CRC diagnosis [101].

High TLR4 expression, especially in CRC cases among adenocarcinoma individuals, is linked to an escalated risk of disease advancement, suggesting its utility as an indicator of CRC progression [102]. TLR4 is crucial for dendritic cell activation and enhancing anti-tumor T cell responses through DAMPs released by cancer cells under stress conditions [103]. Tumor cells expressing TLR4 are linked to decreased recurrence rates, while fibroblasts expressing TLR4 are independently associated with higher recurrence rates and shorter overall survival, suggesting fibroblast TLR4 expression as a prognostic marker in CRC [104].

### 3.6. Drug Resistance

Enhancing the prognosis of CRC patients requires a comprehensive understanding of chemoresistance mechanisms. This is imperative given the persistently poor 5-year survival rates attributable to chemotherapy resistance [105]. Chemoresistance in cancer involves intricate interactions between gene regulation and the cellular microenvironment, highlighting the complexity of the molecular mechanisms [106].

TLR4 signaling in tumor cells has been implicated in promoting immune escape and progression, with TLR4-triggered resistance to apoptosis considered a contributing mechanism [52]. The response to TLR4 ligands can be influenced by single nucleotide polymorphisms of TLR4 genes. Notably, the Asp299Gly and Thr399Ile single nucleotide polymorphisms in the human TLR4 gene may impact individual responses to chemotherapy. The Asp299Gly polymorphism, present in approximately 6% of individuals of mixed European descent, can interfere with HMGB1 binding to TLR4, potentially influencing the chemotherapy-induced immune responses [107].

TLR4 stands out as a promising therapeutic focus in CRC treatment, linked with drug resistance mediated by the PI3K-AKT pathway. This pathway plays a crucial role in regulating multidrug resistance in various cancers. In 5-fluorouracil-resistant human CRC SNUC5 cells, excessive PI3K/AKT pathway activation is evident, and inhibiting AKT activation effectively overcomes 5-fluorouracil resistance [106]. Additionally, TLR4-induced phosphorylation of GSK3β and ERK plays crucial roles in regulating cancer cell survival. Targeting TLR4 becomes crucial for addressing drug resistance in CRC patients [108].

Macrophages, crucial immunosuppressive cells within the tumor microenvironment, are pivotal in chemotherapy resistance in CRC. TAMs contribute to immunosuppression, tumor progression, and chemoresistance by releasing various factors. Targeting macrophages, especially promoting the presence of M1 macrophages, holds the potential for regulating chemotherapy resistance. Activation of TLR4 on immune cells has shown efficacy in inhibiting tumor growth. Investigating TLR ligands that activate TAMs presents a promising approach in preclinical cancer models, demonstrating effective antitumor immune responses [109]. Additionally, TLR4 facilitates resistin-triggered ERK activation and NLRP3 upregulation in CRC cells, and inhibiting TLR4 and ERK attenuates these effects, suggesting a potential strategy to enhance the cytotoxic effects of chemotherapy [110]. TLR4 expression is influenced by the mismatch repair status, directly correlating with MLH1 expression. Mismatch repair-deficient CRC patients, showing better prognosis and chemoresistance, exhibit decreased TLR4 expression when MLH1 is silenced in vitro [111].

*Fusobacterium nucleatum* induces chemoresistance in CRC through the upregulation of BIRC3 via the TLR4/NF-κB pathway. *Fusobacterium nucleatum* infection reduces CRC cells’ chemosensitivity to 5-fluorouracil. Elevated *Fusobacterium nucleatum* abundance is associated with chemoresistance in advanced CRC patients undergoing 5-fluorouracil-based adjuvant chemotherapy post-surgery [112]. The bacterium is widespread in CRC specimens from patients experiencing recurrence following chemotherapy, correlating with clinicopathological features. *Fusobacterium nucleatum* promotes CRC chemoresistance through its involvement in the TLR4/MyD88 signaling pathway and the regulation of microRNAs. Understanding and targeting *Fusobacterium nucleatum* and its associated pathways could provide valuable insights for clinical management and improve CRC patient outcomes [113].

### 3.7. EMT/Migration/Invasion/Metastasis

Metastasis stands as the primary cause of mortality among cancer patients [114]. Metastasis involves a complex series of steps. Initially, a small subset of highly invasive cancer cells spread from the primary tumor. These cells then degrade the extracellular matrix, enter the bloodstream or lymphatic vessels, and form colonies in distant organs. Paget’s “Seed and Soil” theory underscores the importance of the microenvironment, where tumor-derived cytokines like vascular endothelial growth factor (VEGF), TNF, and transforming growth factor-β create premetastatic niches. These niches recruit bone marrow-derived cells, facilitating metastasis [115].

Elevated LPS levels in CRC patients contribute to tumor metastasis [116]. PAR2 activation in CRC cells enhances TLR4 release, promoting cell migration [13]. TLR4 inhibition reduces migration and invasion in CRC [43,117]. High expression of TLR4 and MyD88 is linked to liver metastasis and predicts poor prognosis in CRC [118]. Enhanced TLR4 expression post-chemotherapy promotes CRC cell survival and EMT [108]. TLR2/TLR4, CXCR4, and CXCR7 mRNA are significantly overexpressed in metastatic liver tissues [119].

NF-κB is activated by various stimuli and plays a role in metastatic dissemination through the EMT process, promoting migration to distant organs [26]. In colitis-associated carcinogenesis, NF-κB activation via the LPS/TLR4 pathway contributes to CAC metastasis [120]. GSK-3βregulates cellular metabolism and EMT processes, with TLR4-triggered GSK-3β activation promoting colon cancer cell migration and invasion. Gut microbiota influences CRC initiation and progression, mediating the gut microbiota-CRC metastasis imbalance, and serves as both a predictive biomarker and therapeutic target in CRC [53,92].

SR-B1 enhances IFNα response via TLR2/4 activation, eradicating CRC liver metastases through gene therapy with reduced toxicity [121]. Inflammatory microenvironments support tumor migration, with BMI-1 influencing NF-κB signaling as a potential chemopreventive agent against CAC [122]. Dysregulation in CD14/TLR4 antagonism contributes to the transition between normal epithelial and carcinogenesis [123]. LPS-induced TLR4 signaling activates PI3K/AKT, increasing CRC cell adhesiveness and metastatic capacity [124]. HMGB1, released under glucose deprivation, stimulates colonic myofibroblast migration via RAGE/TLR4 activation and works as a potential therapeutic target for inhibiting solid tumor growth [99]. S100A8 promotes CRC metastasis by targeting TLR4/MD2 [115]. TLR4-mediated tumor immune escape contributes to neoplastic transformation and cancer progression, linking chronic inflammatory disorders to cancer [125]. Reducing tumor cell TLR4 expression decreases metastatic tumor burden in steatotic livers in CRC [126].

Surgery-induced inflammation promotes CRC metastasis, with Nox enzymes, implicated in tumor cell metastasis. LPS induces a transient ROS increase, upregulates Nox1/Nox2, and activates the PI3K/Akt pathway, facilitating CRC cell adhesion to collagen I. The LPS-Nox1 redox signaling axis significantly enhances colon cancer cell metastatic potential, underscoring the importance of targeting Nox1 to mitigate CRC metastasis [127]. LPS induces Gal-1 expression, triggering lactate-mediated EMT processes in CRC cells [56]. The TLR4-D299G mutation promotes carcinogenesis in Caco-2 cells and correlates with advanced stages of colon cancer in humans [128]. TCTP promotes CRC metastasis by regulating HMGB1 and NF-κB signaling pathways [129].

## 4. TCMs with Anti-CRC Effects through TLR4 Signaling Pathway

Currently, scholarly investigations are underway to explore TCM products that target the TLR4 signaling pathway as promising therapeutic modalities for addressing CRC. Building upon the previous discussion of TLR4 pathway-related biochemical processes, this section will now summarize current research on TCMs targeting TLR4 signaling for the treatment of CRC. By examining the role of natural products derived from herbal medicine, we aim to highlight the therapeutic potential and mechanisms through which these compounds modulate TLR4 signaling to inhibit CRC progression. This integrated approach will provide a comprehensive understanding of how natural TLR4 inhibitors from herbal sources can be effectively utilized in CRC treatment strategies. Below is a summary of existing TCM products used in targeting TLR4 signaling for CRC treatment.

### 4.1. The Compounds Derived from TCMs with Anti-CRC Effects through TLR4 Signaling Pathway (Table 1)

**Figure 6:**
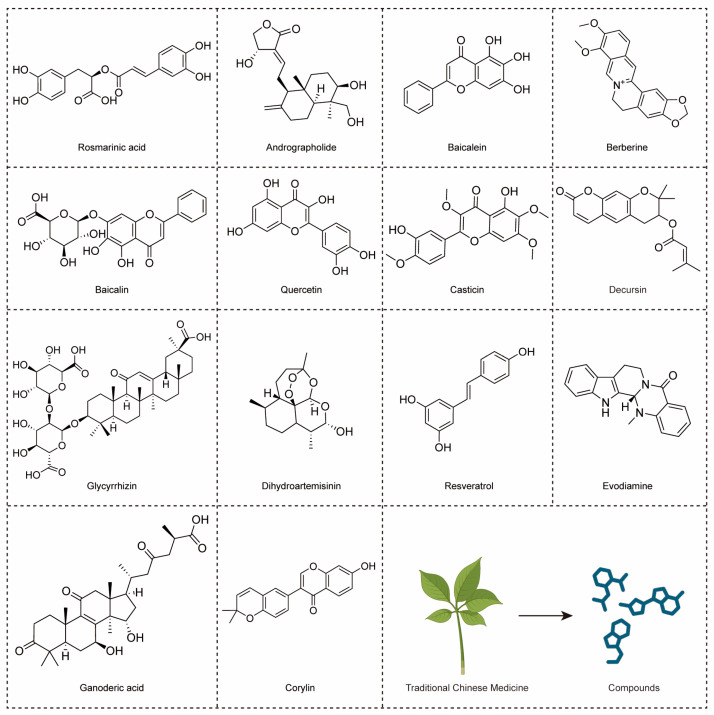
TLR4 signaling inhibitors from TCMs.

The global adoption of TCM for preventing and treating various diseases, including CRC, has surged, owing to its myriad therapeutic properties [20]. Presently, progress in chemical and pharmacological technologies has streamlined the systematic investigation of natural components, providing novel insights into drug discovery [130]. CRC, a significant public health concern in certain regions, is the most prevalent cancer worldwide [131]. Recognizing the challenges associated with resistance and side effects in conventional cancer therapies, numerous studies have shifted focus towards herbal medicines. In the battle against CRC, TCM can profoundly affect the proliferation, apoptosis, migration, and angiogenesis of CRC, offering multifaceted therapeutic potential [132]. More and more research is focusing on the development of TCM products [133]. Additionally, several naturally occurring bioactive compounds isolated from TCMs have been proven to modulate the TLR4 signaling pathway in CRC (Figure 6).

**Table 1:** The compounds derived from TCMs targeting the TLR4 signal pathway in CRC.

Natural Compounds	Sources	Concentration/Dosage	Major Effects	Involved Pathways	Ref.
Andrographolide	*Andrographis paniculata*	in vitro: 20 µM	Inhibiting proliferation and inducing apoptosis of SW620 cells.	TLR4/NF-κB/MMP-9 pathway.	[22]
Resveratrol	*Polygonum multiflorum*	in vitro: 30, 40, 50 mM	Reducing LPS-induced inflammatory responses of Caco-2 and SW480 cell.	/	[23]
Baicalein	*Scutellaria baicalensis*	in vitro: 7.5, 15 µM; 3.125, 6.25, 12.5, 25 µM; in vivo: 10, 20 mg/kg;	Inhibiting proliferation, migration and angiogenesis in CRC.	TLR4/HIF-1α/VEGF pathway.	[25]
Baicalin	*Scutellaria baicalensis*	in vitro: 5–80 μg/mL; 0, 5, 10, 20 μM; in vivo: 20, 40 mg/kg;	Triggering apoptosis and anti-tumor immunity, and inhibiting migration in CRC.	TLR4/NF-κB pathway.	[26]
Berberine	*Coptis chinensis*	in vivo: 50, 100 mg/kg	Regulating short-chain fatty acid metabolism and alleviating the CAC.	TLR4/p-NF-κB p65/IL-6/p-STAT3 pathway.	[24]
Casticin	*Vitex trifolia*	in vitro: 10–100 μM; 40 μM	Inducing G2/M-phase arrest and apoptotic, increasing ROS production and decreasing mitochondria membrane potential and Ca^2+^ of colo 205 cells.	/	[134]
Decursin	*Angelica sinensis*	in vitro: 10 μM	Inhibiting inflammation and metastasis in CRC.	/	[27]
Ganoderic acid	*Ganoderma lucidum*	in vivo: 50 mg/kg	Alleviating chemotherapy-induced fatigue in CRC.	TLR4/Myd88/NF-κB pathway.	[135]
Glycyrrhizin	*Glycyrrhiza uralensis*	in vivo: 15 mg/kg	Inhibiting the inflammation in CRC.	HMGB1/TLR4/NF-κB pathway.	[73]
Quercetin	*Ginkgo biloba*	in vitro: 300, 600 µM	Decreasing chemoresistance in CRC.	TLR4/NLRP3 and ERK/NLRP3 pathway.	[110]
Evodiamine	*Evodia rutaecarpa*	in vitro: 100, 200 µM; in vivo: 10 mg/kg	Inhibiting inflammation in CRC; Inducing G2/M-phase arrest of SW480 cells.	/	[136]
Corylin	*Psoralea corylifolia *	in vivo: 25, 100 mg/kg	Inhibiting inflammation, proliferation of CSCs and colon epithelial cell, improving microbial diversity and community richness, regulating macrophage polarization in CRC.	TLR4/p38/AP-1 pathway.	[137]
Rosmarinic acid	*Perilla frutescens*	in vitro: 25, 50 μM; in vivo: 30 mg/kg	Inhibiting inflammation of CRC.	TLR4/NF-κB/STAT3 pathway.	[100]
Dihydroartemisinin	*Artemisia annua*	in vitro: 1–40 μM; in vivo: 10 mg/kg	Inhibiting inflammation of CRC; Improving cell cycle inhibition and apoptosis in CRC cells.	TLR4 signaling pathway.	[138]

Andrographolide derived from *Andrographis paniculata* (Chuanxin Lian), exhibits antibacterial, anti-inflammatory, and antiviral properties. In SW620 cells, it suppresses proliferation, induces apoptosis, and activates caspase-3 and -9. Its mechanism involves inhibiting of the TLR4/MyD88/NF-κB/MMP9 pathway. Andrographolide shows potential as a CRC treatment by targeting signaling pathways, but requires further validation for efficacy and safety [22]. Resveratrol, found in red wine and grapes, exhibits anti-inflammatory effects. In Caco-2 and SW480 cell studies, resveratrol inhibited iNOS expression induced by LPS and decreased TLR4 expression. Mechanistically, it inhibits IκB phosphorylation and degradation, attenuating inflammatory responses. Resveratrol holds promise for treating IBD, necessitating further investigation of its molecular mechanisms [23]. Baicalein binds to TLR4, inhibiting HIF-1α/VEGF signaling, thus curtailing CRC growth, angiogenesis, and metastasis. Targeted delivery systems like nanoparticles or aptamers could enhance baicalein’s efficacy. This highlights baicalein as a promising therapy targeting TLR4, with potential for treating metastatic CRC [25].

Baicalin from *Scutellaria baicalensis* (Huangqin) possesses anti-cancer, antioxidant, and anti-inflammatory properties. It is safe at therapeutic doses and efficiently inhibits NF-κB, showing potent anti-tumor effects on CRC by suppressing cell viability, inducing apoptosis, and modulating the TLR4/NF-κB pathway [26]. Baicalin’s therapeutic role involves modulating apoptosis and immune response pathways triggered by oxidative stress and inflammation. Key factors in the apoptosis pathway, such as caspase-3, -9, Bax, and Bcl-2, contribute to the suppression of CRC. Additionally, immune regulation involving PD-1/PDL-1 and TLR4/NF-κB is associated with baicalin’s ability to alleviate hepatocellular carcinoma, UC, and CRC [130]. Berberine, a key compound found in medicinal plants like *Coptis chinensis* (Huanglian) or *Cortex phellodendri* (Huangbo), has demonstrated safety and efficacy in reducing CRC recurrence and inhibiting its progression [139]. Berberine also enhances beneficial short-chain fatty acids, reduces fecal LPS content, upregulates intestinal barrier biomarkers, and inhibits the TLR4/NF-κB P65/IL-6/p-STAT3 pathway. Fecal flora transplantation validates its potential clinical application in CRC treatment [24].

Casticin, derived from *Fructus Viticis* (Manjin Zi), effectively inhibits the proliferation of human colon cancer cells. It induces morphological changes, reduces cell viability, and triggers G2/M phase arrest of COLO 205 cells. Moreover, it enhances ROS production, activates caspases, and modulates genes associated with TLR4, cell cycle, apoptosis, and metastasis, suggesting therapeutic potential warranting further in vivo investigation [134]. Decursin and its isomer demonstrate potent anti-tumor, anti-inflammatory, antiangiogenic, and memory-enhancing effects. by inhibiting pro-inflammatory molecules and LPS-induced PRP4 expression. They downregulate TLR4 and NF-κB, offering therapeutic benefits against cancer progression and metastasis-associated morphological changes [27]. Ganoderic acid derived from *Ganoderma lucidum* (Lingzhi), exhibits anti-tumor, anti-inflammatory, and immunomodulatory effects. Co-administration with 5-fluorouracil alleviates muscle fatigue by improving muscle quality and function, enhancing energy metabolism, and reducing inflammation via TLR4/ MyD88/NF-κB pathway inhibition [135].

Glycyrrhizin, a licorice root component, targets HMGB1 signaling, showing anti-inflammatory and anti-carcinogenic properties. Treatment reduces colon tumors, plasma IL-6, and TNF-α. It inhibits inflammation by binding HMGB1, suppresses proliferation and dedifferentiation of CSCs, and DNA damage via the HMGB1/TLR4/NF-κB pathway, suggesting its potential as a CRC preventive agent [73]. Quercetin, a dietary flavonoid abundant in plants, exerts significant anticancer effects, inhibiting cancer cell growth and metastasis. It exhibits low toxicity to normal cells and enhances its inhibitory effects on colon cancer cells when fermented by *Lactobacillus*. Fermented quercetin enhances cytotoxicity and induces cell death when exposed to resistin. Resistin activates ERK phosphorylation via TLR4, upregulating NLRP3 expression in CRC cells, and reducing their sensitivity to 5-fluorouracil treatment. However, L. plantarum fermentation of quercetin mitigates resistin-induced resistance by suppressing NLRP3 expression [110].

Corylin, derived from *Psoralea corylifolia* L. (Buguzhi), displays antioxidant effects and promotes osteoblastic proliferation. It downregulates pro-inflammatory cytokines and inflammation-related markers. Corylin intervention in a colon barrier experiment reduces IEC proliferation and enhances tight junction proteins. Moreover, it alters gut microbiota composition, mitigating colitis-associated cancer risk [137]. Evodiamine, derived from *Evodia rutaecarpa* (Wuzhuyu), displays potent anti-inflammatory and anti-tumor effects. It ameliorates UC symptoms and reduces colonic tumors in CRC murine models. Evodiamine modulates NF-κB and cytokines, inhibiting inflammation and tumor growth. In vitro, it reduces cell viability, induces cell cycle arrest, and impedes NF-κB translocation in colon cancer cells. Molecular dynamics simulations suggest evodiamine’s binding to NF-κB’s ordered domain, highlighting its therapeutic potential for UC and CRC by suppressing inflammation and regulating cytokine secretion [136].

Rosmarinic acid from *Perilla frutescens* (L.) Britt (Zisu) [140] exhibits potent anti-tumor effects in CRC, particularly in CAC. It mitigates colitis severity, inflammation, and tumor development by modulating TLR4-mediated NF-κB and STAT3 activation, suppressing tumor growth factors. In vitro studies reveal its inhibition for the TLR4-MD2 complex, revealing its therapeutic potential of CRC in an inflammatory microenvironment [100]. Dihydroartemisinin, an active metabolite of artemisinin discovered by Nobel laureate Tu Youyou in 1971, displays antimalarial efficacy. It also shows promise in managing inflammatory responses, cancer, and autoimmune diseases. In CAC, dihydroartemisinin inhibits tumor growth by targeting the TLR4 pathway, enhancing cell cycle arrest and apoptosis. Administered throughout the CAC model, dihydroartemisinin improves therapeutic outcomes by reducing inflammation and tumor growth, suggesting its potential for clinical translation in CAC treatment [138].

### 4.2. Formulas of TCMs with Anti-CRC Effects through TLR4 Signaling Pathway

Building on the discussion of individual compounds derived from TCMs targeting CRC via the TLR4 signaling pathway, this section now delves into TCM formulas (Table 2).

*Asparagus racemosus* (Tianmendong), a traditional Chinese herb, displays anti-inflammatory, antioxidant, hypolipidemic, immunomodulatory, and antifungal attributes. Asparagus polysaccharide, one of its constituents, demonstrates cancer therapeutic potential by inducing apoptosis in MDSCs via the TLR4/NF-κB pathway. This effect enhances antitumor immune responses, suggesting Asparagus polysaccharide acts as a promising agent in cancer therapy [46]. *Arctiumlappa* root (Niubang) activates TLR4 signaling, enhancing apoptosis, and reducing cancer cell attachment. It exhibits potent cytotoxicity against cancer cells, modulating TLR4 signaling molecules and suggesting immunomodulatory potential. Future in vivo studies are warranted to explore its efficacy in cancer therapy, and its ability to activate TLR4 downstream pathways and inhibit tumor cell adhesion [133].

Polysaccharides from *Atractylodes macrocephala* (Baizhu) boost immunity by promoting lymphocyte proliferation and activating immune cells. These polysaccharides, abundant in bioactive compounds, regulate immune function through anti-stress and antioxidant pathways. Through activation of the TLR4/MyD88-dependent cascade, they elicit macrophage stimulation, initiating an immune response against cancer. The modulation of the TLR4 pathway regulates innate immunity in CRC cells [141]. *Curcumae longae Rhizoma* (Jianghuang), derived from Turmeric, is a safe FDA-approved herb listed in the Chinese Pharmacopoeia. It is used for ailments like diabetes and arthritis, extract reverses 5-fluorouracil resistance in CRC. It inhibits proliferation and induces apoptosis in resistant cells by targeting the TLR4/PI3K/Akt/mTOR pathway, suggesting its potential as an adjuvant for enhancing 5-fluorouracil efficacy in CRC treatment [106].

*Ganoderma lucidum* (Lingzhi), contains polysaccharide, renowned for its immunoregulatory, hypoglycemic, antioxidant, and anti-cancer effects. It alleviates AOM/DSS-induced colitis and tumorigenesis in mice by modulating gut microbiota, colonic integrity, and immune cell function. In vitro, it exhibits anti-inflammatory effects via TLR4/MyD88/NF-κB and MAPK pathways. Further research is warranted for CRC applications [142]. Polysaccharides from *Lentinula edodes* (Xianggu), like lentinan and MPSSS, have shown health benefits. Lentinan acts as a chemotherapy adjuvant, inhibiting tumor metastasis. MPSSS inhibits lymph angiogenesis in CRC via the TLR4/JNK pathway [143]. Lentinan is effective in treating IBD and CAC by inhibiting TLR4/NF-κB signaling and restoring intestinal microbiota [144]. It reduces tumor numbers, and inflammation, and regulates cancer markers in CAC models, showing promise for IBD and CAC treatment.

Yiyi Fuzi Baijiang powder offers significant benefits in CRC treatment. A study through network pharmacology, explored Yiyi Fuzi Baijiang powder’s effects on CRC, revealing active ingredients. They potentially inhibit spermine oxidase expression, demonstrating anti-inflammatory properties and modulation of the TLR4/NF-κB pathway, vital for future anti-tumor research [146]. Irinotecan, utilized in advanced CRC treatment, leads to intestinal inflammation-related injury. The Shubu Wenshen Guchang recipe demonstrates a significant protective effect against Irinotecan-induced delayed-type diarrhea. This protective mechanism likely entails suppressing the TLR4/NF-κB signaling cascade in the intestines [145].

Sanwu Baisan Decoction demonstrates potent anti-CRC efficiency in mice with CRC. This effect is likely achieved through multiple mechanisms, including the promotion of anti-tumor immune cytokine secretion, induction of cancer apoptosis, maintenance of gut microbiota, and inhibition of tumorigenesis by suppressing the TLR4/COX-2/PGE-2 pathway [48]. In cancer patients with depression, Xiaochai Hu Decoction effectively alleviates depressive symptoms, reduces inflammation, and addresses gut dysbiosis. Animal studies demonstrate tumor inhibition and antidepressant effects. Clinical trials show that Xiaochai Hu Decoction partially restores gut dysbiosis, especially by reducing *Parabacteroides*, *Blautia*, and *Ruminococcaceae* levels. In a CRC xenograft model, Xiaochai Hu Decoction suppresses tumor progression via gut microbiota modulation, implicating the TLR4/MyD88/NF-κB pathway. These findings highlight the therapeutic potential of Xiaochai Hu Decoction in cancer-related depression and emphasize the gut microbiota’s role in cancer management [147].

### 4.3. The Synergistic Effects of TCM Production with Conventional CRC Therapies

Building on our exploration of TCM productions targeting CRC through the TLR4 signaling pathway, we now turn our attention to their synergistic effects with conventional CRC therapies and we aim to uncover the potential synergies that can optimize CRC management strategies. Based on the literature review, the significance of the synergistic effects of these TCMs and their derivatives with conventional CRC treatments primarily lies in four key areas: enhancing efficacy, reducing side effects, combating chemoresistance, and alleviating toxicity.

#### 4.3.1. Enhanced Efficacy

Herbal medicine has been demonstrated to enhance therapeutic efficacy [20]. A meta-analysis on the combined use of oxaliplatin chemotherapy and TCM for palliative care in CRC revealed significant benefits [148]. Andrographolide Scutellarin enhanced the efficacy of 5-fluorouracil in treating CRC while reversing resistance to 5-fluorouracil in patients with colon cancer [149,150,151]. Andrographolide synergistically enhances the antitumor effect of oxaliplatin on CRC cells [152], while also enhancing radiosensitivity [153]. Resveratrol enhances the cytotoxic effect of 5-fluorouracil on colorectal tumors [154]. Berberine enhances irinotecan-induced apoptosis in CRC cells [155].

Quercetin induces cell cycle arrest and apoptosis in CD133(+) cancer stem cells from human CRC cells (HT29), enhancing the anticancer effect of irinotecan [156]. Quercetin enhances the anti-colorectal tumor effects of 5-fluorouracil [157,158,159]. Additionally, fermented quercetin also augments the cytotoxicity and efficacy of the anticancer drug 5-fluorouracil [110]. Compared to using oxaliplatin alone to treat human CRC cells (HT29), combined treatment with quercetin and oxaliplatin synergistically inhibits glutathione reductase activity, reduces intracellular glutathione levels, increases reactive oxygen species production, and decreases cell viability, thereby enhancing the anticancer efficacy of oxaliplatin [160]. Pre-treatment with quercetin significantly enhances cisplatin-induced apoptosis in HT-29 cells, thereby improving the anticancer function of cisplatin during CRC chemotherapy. In addition to enhancing chemotherapy sensitivity, resveratrol treatment also effectively increases the radiosensitivity of CRC by downregulating aberrant angiogenesis-related signaling pathways and promoting radiation-induced lesion formation, rendering colorectal cells more sensitive to radiotherapy [161].

Dihydroartemisinin synergistically enhances the anticancer activity of oxaliplatin in CRC cells [162]. Ganoderma lucidum enhances the effects of 5-fluorouracil in both in vitro and in vivo CRC models, increasing the survival rate of treated mice and reducing tumor volume [163].

#### 4.3.2. Reduced Side Effects

Compared to chemotherapy alone, the combination of TCM and 5-fluorouracil chemotherapy reduces chemotherapy-related diarrhea by 57% and leukopenia by 66% [164]. 5-fluorouracil is one of the most used first-line chemotherapy drugs for treating colon cancer, often causing fatigue and loss of appetite, which may persist for several years post-treatment. This greatly diminishes patients’ quality of life and weakens physical and social functions, leading to treatment restrictions and increased morbidity rates. Ganoderic acid alleviates peripheral and central fatigue induced by 5-fluorouracil in tumor-bearing mice [135]. Ganoderic acid prevents cognitive impairment in mice treated with 5-fluorouracil by preventing mitochondrial damage and enhancing neuronal survival and growth [165].

Irinotecan is a topoisomerase I inhibitor primarily used to treat advanced CRC. Irinotecan and its active metabolite SN-38 directly damage intestinal mucosal cells, which can be restored by the Shubu Wenshen Guchang recipe [145]. Berberine ameliorates gastrointestinal inflammation induced by 5-fluorouracil and irinotecan, including nausea, vomiting, and diarrhea [166,167]. Resveratrol attenuates 5-fluorouracil-induced inflammation in HT-29 CRC xenograft nude mice [168]. Oxaliplatin is an effective anticancer drug used for treating CRC, associated with severe dose-limiting side effects such as peripheral neuropathy. Baicalein neutralizes oxaliplatin-induced neuroinflammation and significantly prevents oxaliplatin-induced sensory nerve conduction defects in rats but does not alter the cytotoxicity of oxaliplatin in HCT-116 cell lines [169].

Quercetin alleviates 5-fluorouracil-induced resistance [170]. Combined with radiotherapy, quercetin enhances tumor radiosensitivity in vitro and in vivo [171]. Dihydroartemisinin combined with capecitabine enhances anticancer efficacy and alleviates capecitabine-induced diarrhea, immunosuppression, and inflammation [172], and additionally synergistically enhances the cytotoxicity of oxaliplatin in colon cancer [173]. Atractylodes macrocephala used in conjunction with oxaliplatin for CRC treatment alleviates oxaliplatin-induced nausea, vomiting, and neurotoxicity [174,175]. Xiaochai Hu decoction mitigates delayed-onset diarrhea induced by irinotecan by increasing beneficial gut flora abundance and reducing inflammation [176].

#### 4.3.3. Inhibition of Chemoresistance

Chemoresistance diminishes the efficacy of chemotherapy, becoming a bottleneck in improving the survival rates of cancer patients, and various natural plant extracts have been reported to possess the ability to reverse cancer resistance [106]. Resveratrol, through modulation of multiple signaling pathways and transcription factors, synergizes with various chemotherapy agents, rendering CRC cells sensitive to chemotherapy drugs, including 5-fluorouracil, cetuximab, irinotecan, and oxaliplatin [168]. Quercetin inhibits NLRP3 inflammasome activation, thereby enhancing the sensitivity of CRC to anticancer drugs [110].

5-fluorouracil is a critical component of systemic chemotherapy for colon cancer, and its use often leads to resistance. However, curcumin has been shown to reverse this resistance [106]. Cetuximab is an EGFR-targeting inhibitor, and resistance induced by KRAS mutations limits its anticancer efficacy; Nevertheless, combined treatment with cetuximab and andrographolide reduces the growth of KRAS-mutant tumors and lung metastases in vivo. Thus, baicalein sensitizes KRAS-mutant tumors to cetuximab [177]. Dihydroartemisinin reduces oxaliplatin resistance [178] and enhances the antitumor activity of 5-fluorouracil against resistant colon cancer cell lines [179]. Furthermore, *Curcumae longae Rhizoma* reverses 5-fluorouracil resistance in CRC cells [106].

#### 4.3.4. Reducing Toxicity

In clinical practice in China, the majority of CRC cases employ TCM in conjunction with conventional treatments, which can reduce the toxicity of chemotherapy or radiotherapy [180]. Combination therapy of quercetin effectively enhances the anticancer function of 5-fluorouracil in HT-29 cells. Compared to the control group, this combination exhibits stronger anti-proliferative and pro-apoptotic effects. This phenomenon is attributed to the inhibition of angiogenesis and vascularization. This combination modulates the apoptotic pathway and minimizes the cytotoxic effects of 5-fluorouracil [161]. Cisplatin is one of the most widely used antitumor drugs for treating colon cancer, but its major dose-limiting drawback is nephrotoxicity. Co-administration of resveratrol with cisplatin can ameliorate cisplatin-induced nephrotoxicity and enhance its antitumor activity [181]. Oxaliplatin, a platinum-based compound, is extensively used for treating certain solid tumors, particularly CRC. Yet oxaliplatin-related neurotoxicity is a major dose-limiting factor. Resveratrol reduces clinical manifestations of oxaliplatin-induced neurotoxicity and hepatotoxicity [182]. Rosmarinic acid mitigates mitochondrial dysfunction and spinal glial activation in oxaliplatin-induced peripheral neuropathy [183].

### 4.4. The Safety/Toxicity of These TCM Compounds

Transitioning from exploring the efficacy and synergistic effects of TCM compounds in CRC treatment, we now delve into a crucial aspect: the safety and toxicity profiles of these compounds. Understanding the safety profiles of TCM compounds is pivotal for their clinical application and integration into CRC management protocols. In this section, we aim to review the existing literature on the safety and toxicity of TCM compounds, offering insights into their potential risks and benefits.

The toxicity and safety of a drug are the primary factors to consider when evaluating its efficacy. The toxic side effects associated with these TCM compounds are documented in Table 3. Andrographolide can reduce sperm count in SD rats and disrupt cell cytoskeleton reorganization. It also impairs the meiotic maturation of mouse oocytes. It induces nephrotoxicity and causes symptoms like lower back pain and nausea. Dosage and duration of administration appear to influence its toxicity. In clinical trials, it has led to mild rash, taste disturbance, headaches, and fatigue [184]. VigiBase (https://www.vigiaccess.org/, accessed on 2 May 2024) is the WHO global database of adverse event reports for medicines and vaccines. In VigiBase andrographolide has three potential side effects, including gastrointestinal disorders (i.e., feces discolored), general disorders and administration site conditions (i.e., chest pain), and immune system disorders (i.e., anaphylactic reaction).

High doses of resveratrol inhibit P450 enzymes, damage DNA, and induce testicular injury, kidney problems, and heart inflammation in rats. It also enhances methotrexate absorption, increases warfarin’s anticoagulant effect, and causes liver toxicity and ulcer damage. Additionally, it can lead to mild gastrointestinal symptoms and hypersensitivity reactions. However, high concentrations may be beneficial in cancer treatment [185]. In VigiBas resveratrol has 56 potential side effects, such as eye disorders (i.e., visual impairment), gastrointestinal disorders (e.g., diarrhea, gastrointestinal disorder, and others), and metabolism and nutrition disorders (i.e., hypoglycemia, abnormal weight gain).

In a study with 68 healthy subjects, single doses of baicalein (100–800 mg) led to elevated C-reactive protein and triglyceride levels, as well as proteinuria. Another trial reported adverse effects such as abdominal pain, constipation, and increased liver enzyme levels with multiple doses of glycyrrhizin (200–800 mg twice daily) in healthy volunteers [186].

Berberine demonstrates minimal toxicity and adverse effects in animal studies. Clinical research on its safety in humans reports only mild gastrointestinal reactions such as diarrhea and constipation. However, there are concerns about exacerbating jaundice in newborns with glucose-6-phosphate dehydrogenase deficiency [187]. Berberine stimulates the uterus, cautioning against its use during pregnancy due to potential fetal harm and transfer through breast milk, warranting careful consideration during breastfeeding [188]. In VigiBase berberine has 142 potential side effects, such as blood and lymphatic system disorders (i.e., hemolysis, thrombocytopenia), ear and labyrinth disorders (i.e., tinnitus), eye disorders (i.e., conjunctival hemorrhage, eye discharge).

Dihydroartemisinin adversely affects the maturation of porcine oocytes during meiosis [189]. In VigiBase dihydroartemisinin has 45 potential side effects, such as ear and labyrinth disorders (i.e., vertigo, tinnitus), eye disorders (i.e., eyelid ptosis, visual impairment), gastrointestinal disorders (e.g., vomiting, nausea, abdominal pain).

Glycyrrhizin has not yet been found to have toxic side effects in the literature, but in VigiBase glycyrrhizin has 380 potential side effects, such as blood and lymphatic system disorders (e.g., thrombocytopenia, anemia, and others), cardiac disorders (e.g., palpitations, cardiac flutter, and others), ear and labyrinth disorders (e.g., tinnitus, vertigo, and others).

Quercetin is considered to be safe [190,191]. But in VigiBase, quercetin has 135 potential side effects such as blood and lymphatic system disorders (i.e., red blood cell abnormality), cardiac disorders (i.e., tachycardia, palpitations, and angina pectoris), ear and labyrinth disorders (i.e., ear pruritus, tinnitus).

**Table 3:** The safety/toxicity of these TCM compounds.

Natural Compounds	Toxicity or Side Effects	Potential Side Effects in VigiBase	Ref.
Andrographolide	Reproductive toxicity, nephrotoxicity, taste disturbance, headache, fatigue, and diarrhea, anaphylactic reaction	Gastrointestinal disorders, general disorders and administration site conditions	[184]
Resveratrol	Reproductive toxicity, cardiac toxicity, nephrotoxicity, increased hepatotoxicity, risk of bleeding and anaphylactic reaction	Eye disorders, gastrointestinal disorders, metabolism and nutrition disorders, etc.	[185]
Baicalein	Elevated levels of C-reactive protein and triglycerides, elevated levels of alanine aminotransferase and aspartate aminotransferase, proteinuria and abdominal pain, constipation	/	[186]
Baicalin	/	/	/
Berberine	Diarrhea and constipation, exacerbation of jaundice in neonates with glucose-6-phosphate dehydrogenase deficiency and uterine stimulation	Blood and lymphatic system disorder, ear and labyrinth disorders and eye disorders, etc.	[187]
Casticin	/	/	/
Decursin	/	/	/
Ganoderic acid	/	/	/
Glycyrrhizin	/	Blood and lymphatic system disorders, cardiac disorders and ear and labyrinth disorders, etc.	/
Quercetin	/	Blood and lymphatic system disorders, cardiac disorders and ear and labyrinth disorders, etc.	/
Evodiamine	/	/	/
Corylin	/	/	/
Rosmarinic acid	/	/	/
Dihydroartemisinin	Impairment of oocyte maturation	Ear and labyrinth disorders, eye disorders and gastrointestinal disorders, etc.	[189]

## 5. Conclusions

CRC represents a complex and serious systemic ailment with diverse etiologies, posing a significant threat to health. Inherent heterogeneity among CRC patients and within tumors contributes to an insufficient benefit from current treatments for a subset of individuals. Consequently, a priority lies in investigating the signaling pathways of CRC cells and identifying new diagnostic/prognostic biomarkers to improve our comprehension of the molecular mechanisms driving CRC occurrence, progression, metastasis, and treatment resistance. TLR4, implicated in various diseases and crucial in intestinal homeostasis, is increasingly associated with aberrant activation in CRC. Clinical trials in CRC treatment have witnessed a rising presence of TLR4-targeting small molecules and biologics, underscoring the attractiveness of TLR4 signaling as a therapeutic target. The establishment of precise targets and the development of safe and effective drugs lay the foundation for subsequent clinical trials, determining optimal dosages and administration schedules. TCM, with a history spanning millennia, is an integral choice for CRC treatment due to its minimal side effects, widespread availability, and cost-effectiveness. This review provides a comprehensive review of the role of the TLR4 signaling pathway in CRC, covering its significance in inflammation and cancer, as well as the potential applications of traditional Chinese medicine in regulating the TLR4 signaling pathway. The research delves into the biochemical processes of the TLR4 signaling pathway, explores its mechanisms in the development of CRC, and conducts detailed analyses of the effects of natural products that affect this pathway.

Various experiments, including X-ray diffraction [192,193], solution NMR [194], electron microscopy [195], site-directed mutagenesis [196], and molecular simulations (MD) [197,198], have been conducted to determine the active/inactive structures of TLRs. MD simulations have been extensively used to model the binding process between TLR4 and its ligands, reveal conformational changes, and simulate interactions with potential inhibitors [199]. Ligands initially bind to the extracellular leucine-rich repeat (LRR) domain of TLRs, triggering intracellular conformational changes that transmit signals [200]. Allosteric effects refer to the influence exerted by ligand binding on a protein or receptor molecule, aiding in understanding protein structure–function relationships and drug design. TLR structures have complex effects on signal transduction, dictating ligand binding, pathway initiation, and efficiency through their interactions with co-factors and cellular localization [201].

**Figure 7:**
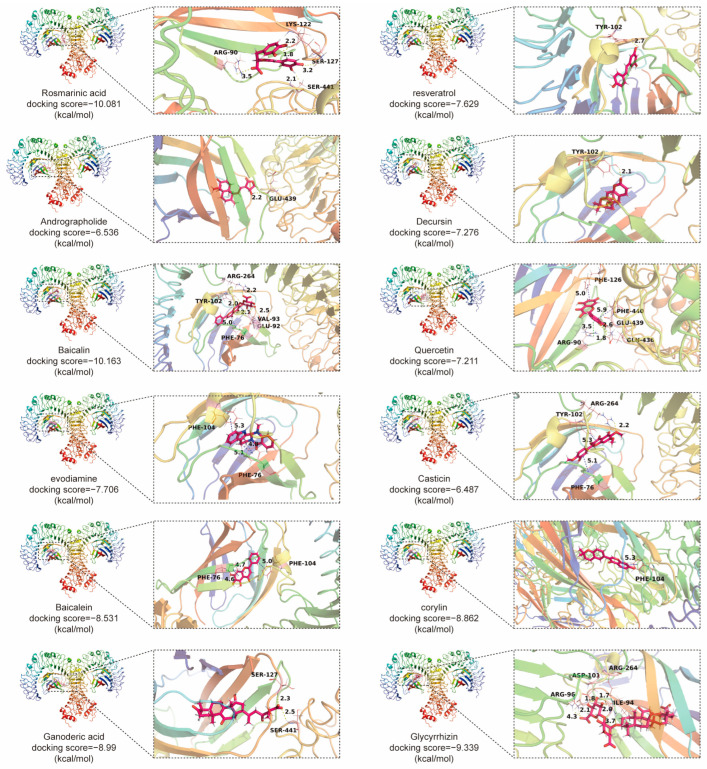
Complex structure of TLR4-MD2 (PDB ID: 4G8A) complex and TLR4 signaling inhibitors from TCMs.

Looking ahead, considering the distinctive role of the TLR4 pathway in CRC, there is a need for the development of more specific inhibitors. The complex nature of TCM, characterized by multiple components and targets [21], poses a challenge in the research and development of TLR4 signaling checkpoint inhibitors. Over the past decade, attention has been focused on how these compounds inhibit CRC by suppressing proliferation, migration, invasion, and inducing apoptosis and autophagy. Molecular docking and computational analyses have identified natural compounds with strong binding to TLR4-MD2 (Figure 7), indicating their potential as TLR4 signaling inhibitors. It is widely recognized that the connection between LPS and TLR4 initially requires the formation of a complex with MD-2 on the cell membrane surface, followed by the attachment of LPS into the hydrophobic pockets of MD-2 and TLR4, thereby triggering downstream inflammatory signaling responses within the membrane. In this study, the complex formed by TLR4 and MD-2 was selected as the docking target. Various active small molecules from TCMs were simulated for their binding affinities within the hydrophobic pockets formed by MD-2 and TLR4 using the Schrödinger molecular docking software (Version 13.1.141). The results indicated that these small molecules primarily bind to the pockets through hydrogen bonds and π-π stacking interactions. This review aims to promote further research and clinical applications of these natural compounds in CRC treatment. The use of traditional Chinese medicine is based on the experience of traditional Chinese medicine practitioners. Although traditional Chinese medicine is generally associated with minimal adverse effects, its direct application without modern clinical research remains a concern. While the study suggests potential applications of natural products in the treatment of CRC, further clinical trials and translational research are needed to validate their safety and efficacy. Traditional Chinese medicine involves multiple molecules and targets, and elucidating its mechanisms is crucial for its global advancement, which remains a challenging and ongoing endeavor.

## Figures and Tables

**Table 2:** The formulas of TCMs targeting the TLR4 signal pathway in CRC.

Full Official Name	Concentration/Dosage	Major Effects	Involved Pathways	Ref.
*Asparagus* polysaccharide	in vitro: 0.25, 0.5 mg/mL	Inhibiting MDSC activity in CRC.	/	[46]
*Arctiumlappa* root	in vitro: 4 mg/mL	Increasing apoptosis in CRC; Decreasing cancer cell attachment to the surface of CRC.	TLR-4/AKT/ERK pathway.	[133]
*Atractylodes macrocephala* polysaccharides	in vitro: 50, 100, 200, 400 μg/mL	Enhancing the phagocytosis of BMDMs by CRC cells.	TLR4/MyD88 pathway.	[141]
*Curcumae longae Rhizoma*	in vivo: 500 mg/kg	Reversing the 5-Fluoruracil resistance in SW480 cells.	TLR4/PI3K/Akt/mTOR pathway.	[106]
*Ganoderma lucidum* polysaccharide	in vivo: 200, 300 mg/kg	Inhibiting inflammation and tumorigenesis in colon.	TLR4/MyD88/NF-κB pathway.	[142]
*Lentinus edodes* Polysaccharides	in vitro: 30 µg/mL; 0.5, 1, 2 mg/mL in vivo: 30 mg/kg; 5, 10, 20 mg/kg	Inhibiting lymphangiogenesis and metastasis of lymphatic, and inflammation in CRC.	TLR4/JNK and TLR4/NF-κB pathway.	[143,144]
Shubu Wenshen Guchang recipe	in vivo: 100, 200 mg/kg	Inhibiting intestinal damage in CRC.	TLR4/NF-κB pathway.	[145]
Yiyi Fuzi Baijiang powder	/	Inhibiting inflammation in CRC.	TLR4/NF-κB pathway.	[146]
Sanwu Baisan Decoction	in vitro: 5.647 mg/mL in vivo: 5, 10, 50 mg/kg	Remodeling gut microbiota and inducing apoptosis in CRC.	TLR-4/COX-2/PGE-2 pathway.	[48]
Xiaochai Hu Decoction	in vivo: 10.27, 20.54 mg/kg	Reducing depressive symptoms and reversing gut dysbiosis in CRC	TLR4/MyD88/NF-κB pathway.	[147]

## Data Availability

No new data were created or analyzed in this study. Data sharing is not applicable to this article.

## Abbreviations

ACAT1	Acyl coenzyme A cholesterol acyltransferase 1
ADAM	A disintegrin and metalloproteinase
AKT	Protein kinase B
AOM	Azoxymethane
CAC	Colitis-associated colon cancer
COX	Cyclooxygenase
CRC	Colorectal cancer
CSCs	Cancer stem cells
CXCR7	CXC chemokine receptor 7
DAMPs	Damage-associated molecular patterns
DCs	Dendritic cells
DSS	Dextran sodium sulfate
EGFR	Epidermal growth factor receptor
EMT	Epithelial–mesenchymal transition
ERK	Extracellular regulated protein kinases
Gal-1	Galectin-1
GSK-3β	Glycogen synthase kinase-3β
HMGB1	High-mobility group box 1
HSP110	Heat shock protein-110
IBD	Inflammatory bowel disease
IECs	Intestinal epithelial cells
IL	Interleukin
IRAKs	IL-1 receptor-associated kinases
IRF	Interferon Regulatory Factor
JNK	c-Jun N-terminal
LPS	Lipopolysaccharide
MANs	Monophosphoryl lipid A- assembled nanovaccines
MAPK	Mitogen-associated protein kinase
MD2	Myeloid differentiation protein-2
MDSCs	Myeloid-derived suppressor cells
MIF	Macrophage inhibitory factor
MyD88	Myeloid differentiation primary response gene 88
NF-κB	Nuclear factor kappa-B
NK	Natural killer
PAR2	Protease-activated receptor 2
PGE-2	Prostaglandin E2
PI3K	Phosphatidylinositol-4,5-bisphosphate 3-kinase
ROS	Reactive oxygen species
STAT3	Signal transducers and activators of transcription 3
TAMs	Tumor-associated macrophages
TAK1	Transforming growth factor-βactivated protein kinase 1
TAp63	Transcriptionally active p63
TCMs	Traditional Chinese medicines
THBS2	Thrombospondin 2
TILs	Tumor-infiltrating lymphocytes
TIPE2	The tumor necrosis factor (TNF)-α-induced protein 8-like-2
TLR	Toll-like receptor
TNF	Tumor necrosis factor
TOPORS	TOP1-binding arginine/serine-rich protein
TRAF6	Tumor necrosis factor receptor-associated factor 6
TRAM	TRIF-related adaptor molecule
TIRAP	Toll/interleukin-1-receptor-domain-containing adaptor protein
TRIF	Toll/interleukin-1-receptordomain-containing adaptor inducing interferon-β
VEGF	Vascular endothelial growth factor
